# A Dynamic Energy Budget Approach for the Prediction of Development Times and Variability in *Spodoptera frugiperda* Rearing

**DOI:** 10.3390/insects12040300

**Published:** 2021-03-29

**Authors:** Andre Gergs, Christian U. Baden

**Affiliations:** Bayer AG, Crop Science Division, Alfred-Nobel Straße 50, 40789 Monheim, Germany; christian.baden@bayer.com

**Keywords:** insect rearing, dynamic energy budget (DEB) theory, *Spodoptera frugiperda*, development, temperature, variability

## Abstract

**Simple Summary:**

The fall armyworm *Spodoptera frugiperda* is a moth that is active during the night. Its larvae cause extensive damage to many crops. Laboratory experiments are conducted to find effective ways to control this pest. One important aspect in research, generally, is that experimental results are reproducible. Reproducibility directly depends on the homogeneity of the test material—the fall armyworm larvae, in our case. The more variable the conditions of the larvae in terms of larval stages or sizes, the more variable and less reliable the research results will be. We used a mathematical model to explore the causes for increased variability in the larval development of the fall armyworm. We found that low air temperatures and poor nutrition increase development times and variability compared to higher air temperature settings and good-quality food. This finding helps researchers to adjust rearing temperatures in a way that allows starting experiments with specific larval stages and low variability on time as planned for their high-quality research.

**Abstract:**

A major challenge in insect rearing is the need to provide certain life cycle stages at a given time for the initiation of experimental trials. The timing of delivery, organism quality, and variability directly affect the outcome of such trials. Development times and intraspecific variability are directly linked to the availability of food and to the ambient temperature. Varying temperature regimes is an approach to adapt development times to fulfill experimental needs without impairment of larval quality. However, current practices of temperature setting may lead to increased variability in terms of development times and the frequency of particular life stages at a given point in time. In this study, we analyzed how resource availability and ambient temperature may affect the larval development of the economically important noctuid species *Spodoptera frugiperda* by means of dynamic energy budget modeling. More specifically, we analyzed how rearing practices such as raising of temperatures may affect the variability in larval development. Overall, the presented modeling approach provides a support system for decisions that must be made for the timely delivery of larvae and reduction of variability.

## 1. Introduction

Laboratory experiments are usually designed in a way that allows the study of biological behaviors in response to the variation of one factor only, while keeping the others as constant as possible. Attempts are made to reduce the variability in the initial conditions of the experiments to increase their statistical power. As such, the experimental outcome is directly linked to the rearing conditions of the biological test organisms and their homogenous state.

Due to its widespread and expanding dispersal and its economic and socioeconomic relevance [1], the fall armyworm, *Spodoptera frugiperda* (J.E. Smith) (Lepidoptera, Noctuidae), is an increasingly important laboratory and field model organism in biological and agricultural research. The fall armyworm is native to the Americas, but in the last few years, it has expanded its distribution over large parts of Africa, Asia, and Australia [2]. This polyphagous noctuid species causes major economic damage, as the 350 known host plants species includes many important crops as well, e.g., corn, rice, sorghum, cotton, and many varieties of vegetables [3]. In addition to its polyphagous abilities, the fall armyworm has other properties that make it a severe pest. These include the absence of a diapause, a high reproductive rate, and high migratory abilities [4,5,6].

The artificial rearing of this important pest species is crucial to obtain more information about its biology, behavior, metabolic rate, and all the other puzzle pieces needed for an integrated pest management program [7]. This becomes even more urgent because of the expanding distribution range of *S. frugiperda*; therefore, rearing conditions and artificial diets are the topics of numerous current publications [7,8,9]. The important influence of the temperature regime on the development times of different larval stages was recently shown by Du Plessis et al. [8], as well as Lopez et al. [10] and Montezano et al. [3].

In this paper, we illustrate how resource availability and ambient temperature may affect larval development and variability by means of dynamic energy budget (DEB) modeling. DEB theory is based on the idea that the principle of energy metabolism is largely conserved across species. DEB models can, thus, be applied to simulate the entire life cycle of a wide range of animals [11], including insects [12,13,14]. First proposed by Kooijman [15], DEB theory has previously been applied to analyze the effects of environmental factors on life history processes [16,17], including temperature [18] and food availability in a population context [19,20].

In this paper, we aimed to analyze how resource availability in laboratory cultures and rearing practices, such as growth-controlling temperatures, affect larval development and variability in *S. frugiperda*.

## 2. Materials and Methods

DEB models quantify the rates at which organisms assimilate energy from the environment and subsequently allocate this energy via a reserve compartment to structural growth, the reproductive system, and the maintenance of bodily functions. The models describe the entire life cycle of an organism starting with the embryonic phase to quantify somatic (structural) growth, maturation, and reproduction. Maturity thresholds for birth and puberty mark the onset of feeding and investment of energy into reproduction, respectively. As all modeled rates are dependent on temperature and energy acquisition as a function of food availability, both environmental factors are key drivers in DEB model systems. Many decades of research in DEB theory have led to the development of the Add-my-Pet (AmP) database, where data, models, and sets of parameters for more than 2800 species have currently been collected. Within the collection, the parameter set for a given species is available and can be used in combination with the well-tested model code that is used for the parameter estimation. All of the data used for estimation, as well as the assumptions used to link data and the model, are well documented and openly accessible. Herein, we used a DEB model for *S. frugiperda* previously been developed and published via the AmP collection [21]. The model makes use of the “hex” variant of the DEB system to cover the life cycles of holometabolic insects and some other hexapods. The model covers the morphological life stages of the egg, larva, pupa, and the imago, as well as the functional stages, including the embryo and reproductive stages. As a deviation from the standard model [22], the larval stage accelerates in terms of growth [23] and allocates energy to reproduction. Larval stage transitions are marked by instar-specific stresses, depending on their structural length. For further details of the model implementation and parameters, see [21]. Exemplified code is available from the Supplementary Material S2.

For all scenario analyses and the simulation of developmental variability, we included stochasticity in the model: We multiplied the maximum specific assimilation flux (*p_Am_*), which is one of the primary DEB parameters, with a random factor drawn from a normal distribution with a mean of one. The range of variability for a given setting was explored by means of Monte-Carlo simulations, i.e., repeated simulation runs for different random factors. Note that by posing variability on the parameter *p_Am_* and employing a constant value for the scaled functional response *f*, we affectively assumed that the assimilation efficiency differs among individual larvae.

For model validation, we mimicked the variable laboratory conditions as employed in an independent experiment and compared the model output and data. The culture conditions and experimental set-up were as follows. In *S. frugiperda* rearing, we used cylindrical flight cages (diameter, 24 cm; height, 26 cm) containing 200 adults (male/female ratio, approximately 1:1) fed with a sugar solution. The temperature was 25 °C, humidity 50%, and the flight cages were transparent, allowing exposure to a dimmed natural light regime. Females deposited their eggs in batches on a piece of paper kitchen towel, forming the lid of the cylindrical flight cages. Eggs were allowed to mature and hatch at 26 °C and in darkness in plastic tubs (18 cm length × 13.5 cm width × 6 cm height) containing a layer of artificial diet. At the second larval stage, the larvae were separated into individual Petri dishes (diameter, 5.5 cm) and cultured at 25 °C, 60% humidity, and in darkness until pupation. The recipe for 1 L of the artificial diet was: 0.43 L of hot water to solve 14.5 g of agar and 18 g of alfalfa powder, and then after adding 0.325 L of cold water, the other ingredients, 0.5 mL of rapeseed oil, 40 g of baker’s yeast, 0.7 g of Wesson Salt Mix, 1 g of β-Sistosterol, 0.5 g of L-Leucin, 4 g of ascorbic acid, 3.7 g of vitamin mix, 3.6 g of sorbic acid, 133 g of bean flour, 0.5 g of antibiotics, and 3 mL of 4- hydroxybenzene S were added. The validation experiment was performed using a variable-temperature regime and was started with one of the mass rearing plastic containers derived from the laboratory culture. To prevent cannibalism [24], 36 larvae were separated during the second larval instar into small Petri dishes (5.5 cm in diameter) filled with an ad libitum amount of artificial diet. These Petri dishes were shifted between different temperature regimes using a series of climate cabinets maintained at 22, 26, and 30 °C. The larval stage attained was determined individually once to twice a day.

## 3. Results

**Figure 1:**
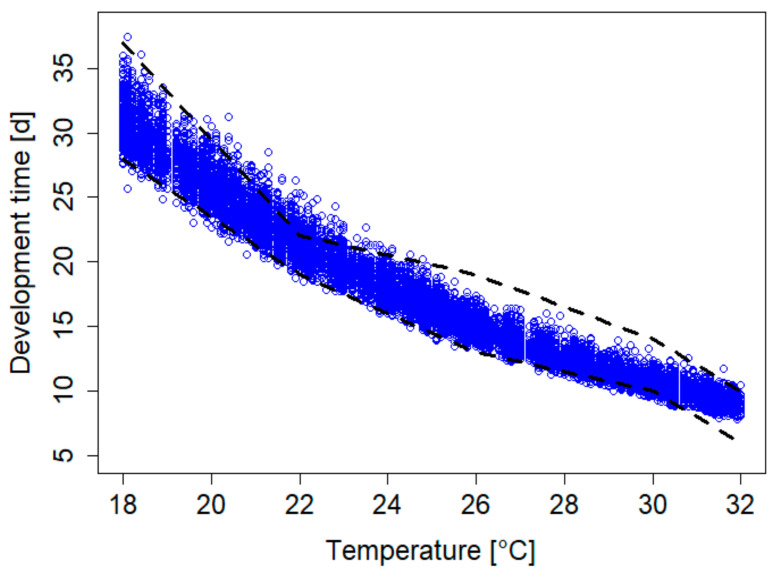
Simulated (dots) and measured range (dashed lines; Du Plessis et al. [8]) of the individual larval development times in days (d) in *S. frugiperda*.

We used a DEB model for *S. frugiperda* to simulate larval development times under different rearing conditions. The simulated larval development times, on average, ranged from 9.0 to 31.2 days at 32 °C and 18 °C, respectively (Figure 1), assuming ad libitum food conditions. In order to account for the variability in development times that were usually observed in laboratory experiments, we estimated a standard deviation in the maximum specific assimilation flux, one of the primary DEB parameters, of 0.087 J d^−1^ cm^−2^, based on the empirical range observed by Du Plessis et al. [8]. The resulting variability in larval development times is shown in Figure 1.

**Figure 2:**
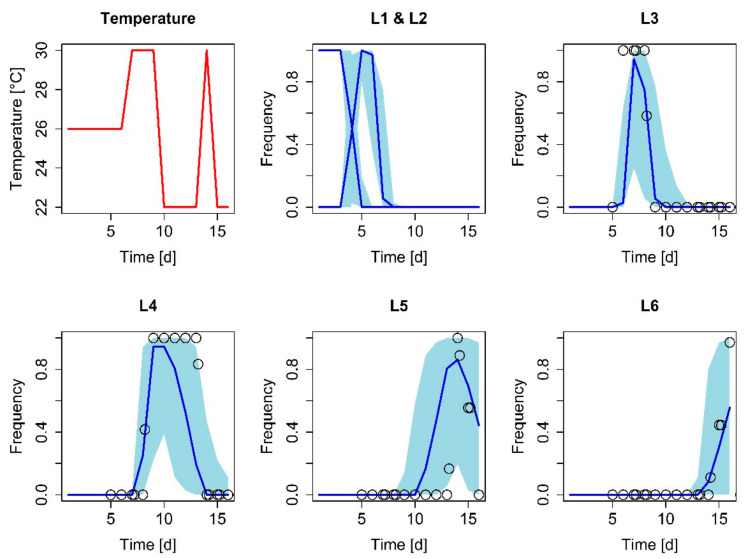
Frequency of the larval instars measured over time in days (d) for an exemplified variable temperature scenario (**red line**). The median and 95% confidence limits of the model prediction are represented by the blue lines and shade areas, respectively. Dots indicate measured data. The instar frequencies were recorded twice per day from instar 3.

For validation, model predictions were compared to experimental data (Supplementary Material S1) that had not been used for model development. In this laboratory experiment, ambient temperature was varied between 22 and 30 °C. Starting from the first instar, under these conditions, all larvae reached the sixth instar within 16 days. The DEB model accurately predicted the larval stage transitions over time—most of the measured data points were within the prediction interval (Figure 2), indicative of a good model performance. Interestingly, the modeled variability increased within the fourth instar, coinciding with a drop in ambient temperature at day 10 of the experiment.

**Figure 3:**
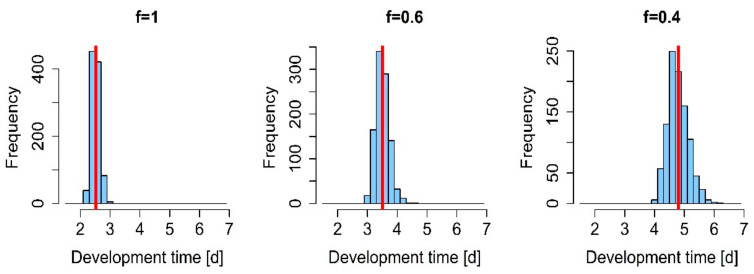
Simulated development times in days (d) (n = 1000) for the example of the fifth larval instar reared at 26 °C and three different feeding regimes: Ad libitum (f = 1), intermediate (f = 0.6), and low (f = 0.4) food availability. Blue columns represent the frequencies of the development times of larvae (n = 1000), and the red vertical line indicates the mean development time per instar.

In the scenario analyses, we subsequently analyzed the variability in larval development times with respect to food availability and fluctuating temperatures in more detail. We varied the value for the scaled functional response in the DEB model to simulate different experimental food conditions and observed the model outputs in terms of development times, starting from the first instar larvae. The scaled functional response can have values between f = 1 (ad libitum) and f = 0 (absence of food) and might represent different food qualities or quantities. The model simulations revealed that variability in larval development times may increase with decreasing food quality or quantity, as illustrated for the fifth instar larvae in Figure 3. Moreover, the simulated development times increased with increasing values for the scaled functional response, as did the standard error (Figure 5).

**Figure 4:**
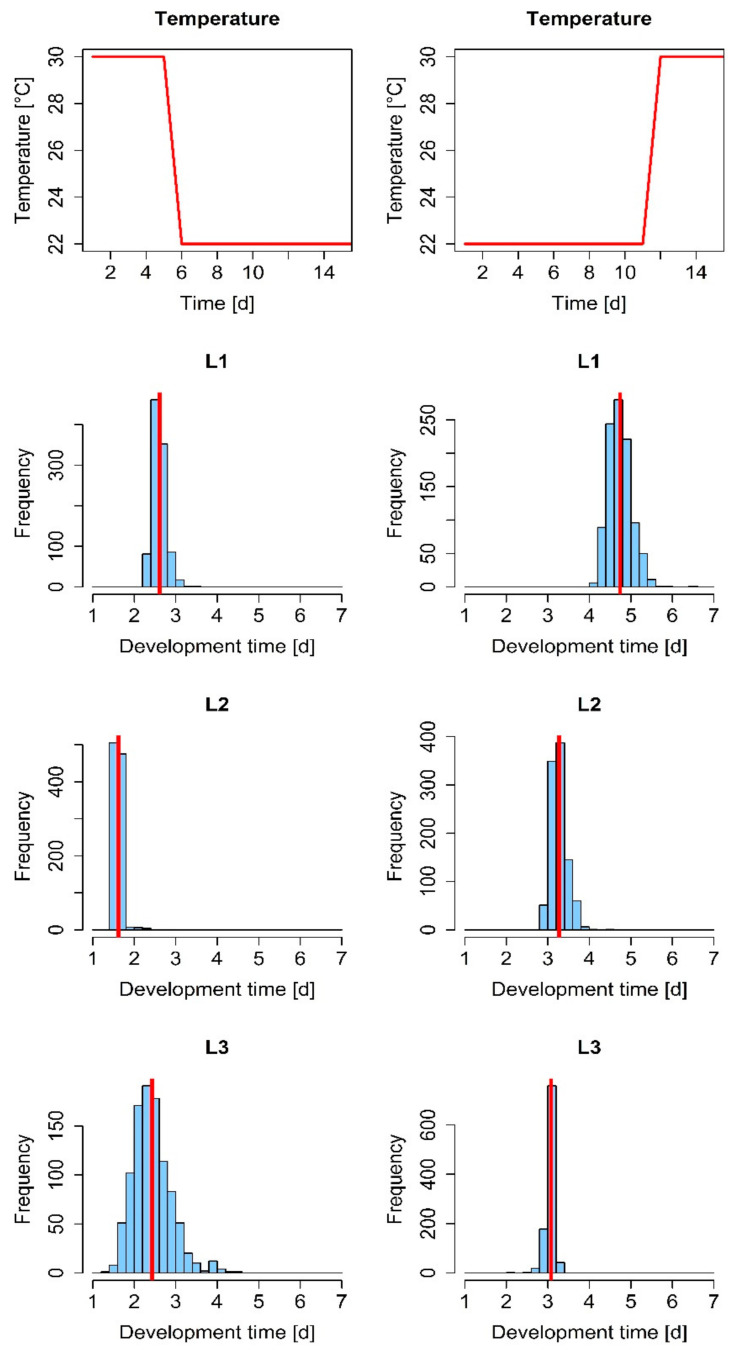
Simulated larval development for decelerating (**left**) and accelerating (**right**) temperature regimes. Blue columns represent the frequencies of the development times of larvae (*n* = 1000), and the red vertical line in the histograms indicates the mean development time per instar.

The simulated larval development times were also affected by up-/downregulation of ambient temperatures (Figure 4). We decreased the simulated temperatures between 30 and 22 °C just before the transition into the third larval instar, and vice versa. This model analysis revealed that the downregulation of ambient temperatures may result in increased variability (Figure 4, left), while the opposite was the case for increasing temperature regimes (Figure 4, right).

## 4. Discussion

The DEB model provides an excellent basis for the prediction of the developmental behavior in *S. frugiperda* rearing, particularly regarding changes in temperature and provision of nutrients. The presented model adequately predicts development times in a changing rearing environment and allows for the analysis of variability among individuals.

Increased variability, with longer development times at lower ambient temperatures, were reported for *S. frugiperda* by Du Plessis et al. [8] (Figure 1) and Garcia et al. [25]. The authors of these studies fitted linear regression models to temperature data for different stages to evaluate the development times and to estimate the temperature thresholds and the number of degree-days. However, Du Plessis et al. [8] argued that the developmental rates become non-linear at unfavorable temperatures. Deviations of the linear temperature model were thus employed, e.g., by Garcia et al. [26], to describe the development rates in *S. frugiperda* beyond empirical observations.

DEB models, by contrast, are based on first principles of bioenergetics. They capture basic life history processes, such as feeding, development, growth, maintenance of bodily functions, reproduction, and senescence under environmental fluctuations in temperature and food availability in one coherent framework and a relatively low number of parameters [11]. The core concept of DEB theory is consistent with the principles of thermodynamics [27,28] and general trends in evolutionary history [29]. The modular structure of DEB models allows specific environmental attributes or stressors to be accounted for without changing the core of the theory. In DEB models, changes in life history trajectories, such as growth and development in response to environmental temperatures, are considered to be a result of changes in bioenergetic rate constants. It has been demonstrated by means of DEB modeling that different life history processes, such as food ingestion, growth, and reproduction, share the same Arrhenius temperature [18]. 

**Figure 5:**
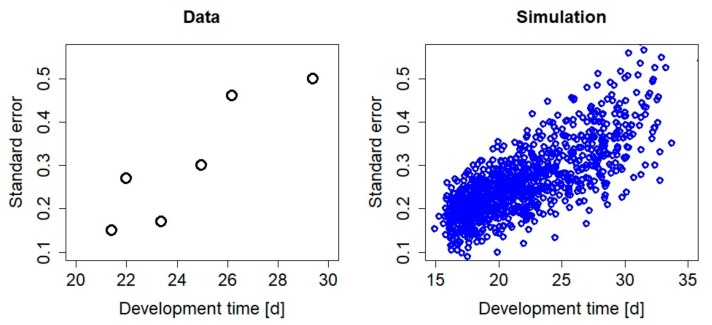
Standard error as function of development time in days (d). The data cover development times (larvae to adults), as measured by daSilva et al. [30], for different food types (soybean, cotton, maize, oats, wheat, and an artificial diet) at an ambient temperature of 25 ± 2 °C. Simulated mean development times (first instar to pupation) and standard errors (n = 10) were calculated 1000 times for random values of the scaled functional response between f = 0.4 and f = 1 and temperatures in the range of 25 ± 2 °C.

In the DEB model, the three-parameter Arrhenius model that was parameterized for *S. frugiperda* [21] describes the non-linear temperature response in larval development times well (Figure 1). Moreover, the same Arrhenius parameter described the instar-specific developmental times well, except for the first instar, where a deviating parameter value was needed [21]. The DEB model approach thus requires fewer parameters and less experimental testing compared to stage-specific linear regression approaches. As regards food, the modeled increase in variability with decreasing values for the scaled functional response f (Figure 3) captures the general pattern of standard errors being increased if development times are prolonged—see, e.g., daSilva et al. [30] for different food types such as oats, cotton, or an artificial diet (Figure 5). Note that simulated development times represent the time span from egg hatching until pupation, while the data [30] also cover the pupa stage, which explains the difference in the development times and the standard errors between the simulation and data. No extra parameters were needed to simulate the different food levels, as the energy acquisition from the environment and subsequent bioenergetic fluxes are inherent properties of the DEB model. 

The DEB model revealed that lower temperatures and food sources of low nutritional value lead to more heterogenous development times (Figure 3 and Figure 4), which subsequently result in more heterogenous colonies or cohorts (e.g., different larval stages). We gained these results both via the model and our experimental data independently of one another (Figure 4). As these effects lead to more homogenous or heterogenous colonies even within a rather short time (Figure 4), every change in a temperature regime should be conducted thoughtfully. For example, the lowering of temperatures overnight (e.g., for slowing down the growth rate to ensure the desired larval stage for a given experiment day) would lead to a higher developmental diversity within the cohort, whereas a higher temperature would have the opposite effect. Therefore, for more homogenous rearing, conditions should include a rather high temperature, between 26 and 30 °C [8], with a good quality food source (Figure 3) [7] to ensure reproducible experimental data with less variability in *S. frugiperda* research. 

If the temperature conditions should change during rearing, the DEB model for *S. frugiperda* is a useful tool to predict and understand the resulting changes in development times and variability. On the contrary, with the help of the model, rearing conditions can be calculated and predicted beforehand, decreasing the need to interfere in running larval rearing. The DEB model can therefore enable researchers to obtain the desired larval stage at a specific date.

A further potential application of the model is for estimating the development rates in field populations of *S. frugiperda*, providing an alternative calculation type besides the degree-days model [8], if there is sufficient information about the temperature and host plant nutrients available (e.g., *S. frugiperda* larval development times on cotton are much longer than on maize [31]). Therefore, DEB models provide important tools for future insect pest management research in laboratory (rearing) and field population biology. They could enhance risk management tools, e.g., for seasonal multigenerational migration, to further improve existing population dynamics and migration models [25,26]. For instance, the NicheMapR package can be used to make field calculations as a function of microclimate using the DEB model [32].

## 5. Conclusions

The dynamic energy budget model is a powerful tool to predict the development times in *S. frugiperda* rearing and can provide guidance on how to adjust the development times to individual needs. Furthermore, it helps to reduce possible side effects by avoiding unnecessary developmental variabilities within a larval cohort caused by unfavorable temperature or nutritional regimes. Many potential interactions with other models predicting population dynamics in the field can be envisaged.

## Data Availability

Validation data (Figure 2) are available in the Supplementary Materials.

## Supplementary Materials

The following are available online at https://www.mdpi.com/article/10.3390/insects12040300/s1: S1: Validation data and S2: model code.
